# Resistin, insulin sensitivity and markers of inflammation in a cohort of Romanian patients under combined antiretroviral therapy

**DOI:** 10.1186/1471-2334-14-S7-P87

**Published:** 2014-10-15

**Authors:** Cătălin Tilişcan, Victoria Aramă, Raluca Mihăilescu, Daniela Munteanu, Mihaela Rădulescu, Adriana Hristea, Cristina Popescu, Ana Maria Tudor, Mihai Lazăr, Roxana Petre, Adrian Streinu-Cercel, Daniela Adriana Ion, Sorin Ștefan Aramă

**Affiliations:** 1 National Institute for Infectious Diseases "Prof. Dr. Matei Balş", Bucharest, Romania; 2 Carol Davila University of Medicine and Pharmacy, Bucharest, Romania

## Background

Several authors have recently reported that resistin, a novel adipokine, may be associated with insulin resistance in HIV patients. Our objective was to evaluate resistin dysfunction in correlation with insulin sensitivity, lipid abnormalities and markers of inflammation in a cohort of adult HIV infected patients who were under complex combined therapy (cART).

## Methods

We performed a transversal study that used the following inclusion criteria: non-diabetic patients with documented HIV infection, undergoing stable cART for at least 6 months. Clinical, metabolic, inflammatory and immuno-virological patterns were assessed (age, sex, body mass index, HIV load, actual and nadir CD4, duration of HIV infection and antiretroviral therapy, lipid panel, C-reactive protein - CRP). Resistin levels were evaluated using KAPME Biosource EASIA. In order to test the sensitivity to insulin we used the QUICKI index, the best surrogate marker after glucose clamp index. Parametric and non-parametric variables were described using means (±Standard Deviation - SD) and medians (Interquartile Ratio - IQR), respectively.

## Results

We enrolled 94 patients (56.4% males, 43.6% females), with a mean age of 31.9 (±13.5) years. The median time from HIV diagnostic was 63 (74) months; the median time of treatment was 60 (50) months. More than half of patients (72.3%) had undetectable HIV load and the median CD4 count was 492 (419)/cmm. The mean level of resistin was 6 (±2.6) ng/mL. The most frequent resistin dysfunction, after adjusting the results by sex and age, was hyporesistinemia (40.2%); hyperresistinemia was less frequent (17.4%). Most patients had insulin resistance (66.3%), based on QUICKI levels below the cut-off point of 0.33. We found no relation between QUICKI values and resistin, in a linear regression model (R=0.054, p=0.614) or correlation between the presence of insulin resistance and resistin dysfunction (p=0.320). Lipid metabolism abnormalities were not influenced by resistin dysfunction. Resistin serum values were positively correlated with the levels of CRP (R=0.21, p=0.05).

## Conclusion

In our cohort of young HIV infected patients, insulin resistance was not mediated by resistin dysfunction, contrary to recent reports, but may contribute to an increased inflammatory profile.

